# Hexose-6-phosphate dehydrogenase controls cancer cell proliferation and migration through pleiotropic effects on the unfolded-protein response, calcium homeostasis, and redox balance

**DOI:** 10.1096/fj.201700870RR

**Published:** 2018-01-08

**Authors:** Maria Tsachaki, Natasa Mladenovic, Hana Štambergová, Julia Birk, Alex Odermatt

**Affiliations:** Division of Molecular and Systems Toxicology, Department of Pharmaceutical Sciences, University of Basel, Basel, Switzerland

**Keywords:** endoplasmic reticulum, NADPH, signaling

## Abstract

Hexose-6-phosphate dehydrogenase (H6PD) produces reduced NADPH in the endoplasmic reticulum (ER) lumen. NADPH constitutes a cofactor for many reducing enzymes, and its inability to traverse biologic membranes makes *in situ* synthesis of NADPH in the ER lumen indispensable. The H6PD gene is amplified in several types of malignancies, and earlier work pointed toward a potential involvement of the enzyme in cancer cell growth. In the present study, we demonstrated a pivotal role of H6PD in proliferation and migratory potential of 3 human breast cancer cell lines. Knockdown of H6PD decreased proliferation and migration in SUM159, MCF7, and MDA-MB-453 cells. To understand the mechanism through which H6PD exerts its effects, we investigated the cellular changes after H6PD silencing in SUM159 cells. Knockdown of H6PD resulted in an increase in ER lumen oxidation, and down-regulation of many components of the unfolded protein response, including the transcription factors activating transcription factor-4, activating transcription factor-6, split X-box binding protein-1, and CCAAT/enhancer binding protein homologous protein. This effect was accompanied by an increase in sarco/endoplasmic reticulum Ca^2+^-ATPase-2 pump expression and an decrease in inositol trisphosphate receptor-III, which led to augmented levels of calcium in the ER. Further characterization of the molecular pathways involving H6PD could greatly broaden our understanding of how the ER microenvironment sustains malignant cell growth.—Tsachaki, M., Mladenovic, N., Štambergová, H., Birk, J., Odermatt, A. Hexose-6-phosphate dehydrogenase controls cancer cell proliferation and migration through pleiotropic effects on the unfolded protein response, calcium homeostasis, and redox balance.

Hexose-6-phosphate dehydrogenase (H6PD) (1) is the only well-characterized enzyme found responsible to date for producing reduced NADPH in the lumen of the endoplasmic reticulum (ER), through conversion of glucose 6-phosphate to 6-phosphogluconate. The same reaction is performed in the cytosol by glucose-6-phophate dehydrogenase (G6PD), representing the first step of the pentose phosphate pathway. In contrast to its cytosolic counterpart, H6PD can also perform the second step of this pathway, acting as a 6-phosphogluconolactonase (2). Because NADPH is regarded to be impermeable through the ER membrane (3), a separate NADPH pool is present within the ER. NADPH is widely used as a cofactor for reducing enzymatic reactions in the cytosol. In contrast, the relevance of luminal NADPH production by H6PD has not been sufficiently explored. The only enzyme that has been undeniably shown to use NADPH as a cofactor in the ER lumen is 11β-hydroxysteroid dehydrogenase type (11β-HSD)-1 (4, 5), catalyzing the oxoreduction of the inactive glucocorticoid cortisone to its potent equivalent cortisol (6). Mice deficient in H6PD (H6PD^−/−^) present with skeletal muscle defects that are independent of 11β-HSD1, indicating unknown functions of H6PD for cell physiology (7, 8). Muscles from H6PD^−/−^ mice compared to wild-type tissues exhibited an increase in 78 kDa glucose-regulated protein (Grp78 or Bip) levels, which is involved in both protein folding and the unfolded-protein response (UPR) pathway. In addition, the levels of the UPR mediator split X-box-binding protein (sXBP)-1 were increased, and microarray analysis showed altered expression of several UPR proteins. Furthermore, muscles from H6PD^−/−^ mice showed decreased expression of the sarco/ER Ca^2+^-ATPase (SERCA), which is responsible for pumping calcium from the cytosol into the ER at the expense of ATP.

Recently, down-regulation of H6PD with short interfering (si)RNAs in the murine cancer cell lines CT26 (colon) and 4T1 (breast) correlated with a reduction in the overall glucose uptake, as well as a decreased cell proliferation (9). In addition, H6PD was identified in a proteome-wide screening as a target of the poly(ADP-ribose) polymerase (PARP) inhibitors rucaparib and niraparib (10). In this report, knockdown of H6PD increased apoptosis in a triple-negative breast cancer cell line (CAL-51), where the researchers observed a concomitant increase in PARP-1 and caspase-3 cleavage. However, these findings could not be reproduced in another triple-negative breast cancer cell line (MDA-MB-468). The results of the above experiments pointed toward a potential role of H6PD in cancer cell physiology and a possible link between H6PD function and energy metabolism. The *H6PD* gene is amplified in 3–4% of pancreatic, sarcomatous, and ovarian tumors and in 1–2% of breast, lung adenocarcinoma, and melanoma tumors, supporting a role of the enzyme in cancer cell growth.

In the present study, we showed that H6PD promotes cancer cell proliferation by using 3 different breast cancer cell models, each representing one of the main molecular subtypes of breast cancer: the triple-negative cell line SUM159 [does not express progesterone receptor (PR^−^), estrogen receptor (ER^−^), nor the Her2 receptor (Her2^−^)], the PR^+^, ER^+^, Her2^−^ cell line MCF7, and the PR^−^, ER^−^, Her2^+^ cell line MDA-MB-453. We further demonstrated that H6PD knockdown dramatically reduces migration in all cell lines tested. Subsequently, we attempted to elucidate the mechanism through which H6PD influences cancer cell growth. Our results suggest a major role of H6PD in regulating UPR signaling proteins, as well as ER calcium balance. H6PD depletion also caused an increase in ER oxidation and a significant decrease in cellular oxygen consumption rate. The above findings highlight, for the first time, the consequences of NADPH depletion within the ER for cancer cell physiology, paving the way for further investigations into the ER-related molecular pathways that promote malignant cell proliferation and migration.

## MATERIALS AND METHODS

### Chemicals

Unless otherwise stated, all chemicals were purchased from Millipore-Sigma (Buchs, Switzerland).

### Cell lines and transfections

The SUM159, MCF7, and MDA-MB-453 cell lines were acquired from American Type Culture Collection (Manassas, VA, USA), tested monthly for mycoplasma contamination, and cultivated under standard conditions (37°C, 5% CO_2_). SUM159 cells were cultured in Ham’s F12 nutrient mixture (Thermo Fisher Scientific, Waltham, MA USA), supplemented with 5% fetal bovine serum (FBS) and 5 μg/ml bovine insulin (cat. no. I6634; Millipore-Sigma) (11–14). MCF7 cells were cultured in DMEM containing 2 mM l-glutamine, 4.5 g/L glucose, 10% FBS, and nonessential amino acid mixture. MDA-MB-453 cells were cultivated in RPMI-1640 medium supplied with 10% FBS. All cell culture media were supplemented with 100 U/ml penicillin, 0.1 mg/ml streptomycin, and 10 mM hydroxyethyl piperazineethanesulfonic acid (HEPES) buffer (pH 7.4).

For siRNA delivery Lipofectamine RNAiMax (Thermo Fisher Scientific) was used. Typically, 50 pM siRNA and 2.5 μl Lipofectamine reagent were used per 300,000 cells. The target sequence of the mock siRNA was 5′-UGGUUUACAUGUUUUCUGA-3′ and of the H6PD siRNA was 5′-GGGCUACGCUCGGAUCUUG-3′ (GE Dharmacon, Lafayette, CO, USA).

Lipofectamine 2000 (Thermo Fisher Scientific) was used for DNA transfection of the SUM159 cell line; 2.5 μg plasmid DNA and 5 μl reagent were used per 180,000 cells, which were seeded the day before transfection. The medium was exchanged with fresh culture medium 6 h after transfection.

### Protein expression analysis

The procedures for cell lysis, protein extraction, and Western blot analysis have been previously described (15). Antibodies against the following proteins were used: H6PD (HPA004824; Millipore-Sigma); protein kinase R-like ER kinase (PERK; 3192 ), eukaryotic initiation factor (eIF)-2α (9722S), phospho-(p)eIF2α (119A11), ATF4 (11815S), ATF6 (65880S), sXBP-1 (12782S), and CCAAT/enhancer binding protein homologous protein (CHOP; 2895S; all from Cell Signaling Technology, Danvers, MA, USA); Grp94 (16) and Grp78 (610978; BD Biosciences, San Jose, CA, USA); protein disulfide isomerase (PDI; Ab2792; Abcam Inc, Cambridge, United Kingdom); ERp44 (17), ERp72 (Stressgen, San Diego, CA, USA); calreticulin (2891S; Cell Signaling Technology), calnexin (SAB4503258; Millipore-Sigma); SERCA2 (cat. no. MA3-919; Thermo Fisher Scientific); actin (sc-1616) and inositol trisphosphate-3 receptor (IP3R)-III (sc-7277; both from Santa Cruz Biotechnology, Dallas, TX, USA); and α-tubulin (GTX628802; GeneTex, Irvine, CA, USA). All antibodies were used at a concentration of 1 μg/ml, except for those against CHOP and IP3RIII, which were used at 2 μg/ml, and those against calnexin, PDI, and α-tubulin, which were used at 0.25 μg/ml. For every sample, 25–30 μg total protein extract was loaded on polyacrylamide gels.

### Real-time quantitative PCR and gene expression analysis

To assess mRNA levels, total RNA was extracted with the TRI Reagent (Millipore-Sigma), and cDNA was subsequently produced by reverse transcription with Maloney murine leukemia virus reverse transcriptase (Promega, Madison, WI, USA). The KAPA SYBR Fast Kit (Millipore-Sigma) was used for quantitative PCR (qPCR) analysis, and the reactions were performed in the Rotor Gene Real-Time Cycler (Corbett Research, Sydney, New South Wales, Australia). Data were normalized to the expression levels of the endogenous control gene peptidyl-prolyl *cis*-trans isomerase A according to the 2^−ΔΔ*C*_*t*_^ method for relative quantification (18). The primers are listed in Supplemental Table 1.

### Real-time cell proliferation assay

For real-time, impedance-based monitoring of cell proliferation, the xCELLigence real-time cell analyzer (RTCA) DP instrument was used (ACEA Biosciences Inc., San Diego, CA, USA). Cells were transfected with siRNAs or left untreated and seeded on an E-Plate View 16 featuring microelectrode sensors. After preliminary evaluation of optimal cell density, 5,000, 7,500, and 10,000 cells were seeded per well, of the SUM159, MCF7, and MDA-MB-453 cell lines, respectively. Quadruplets of every sample were prepared, and signal detection was registered every 30 min over a period of 96 h. Data analysis was performed with the RTCA software 2.0.

### High-content imaging

After siRNA transfection on 96-well plates, cells were fixed at the indicated time points with 4% formaldehyde for 12 min and washed 3 times with PBS; the nuclei were stained with 5 μg/ml Hoechst-33342 (Thermo Fisher Scientific) for 15 min. After 4 washes with PBS, the number of nuclei was quantified with the ArrayScan high-content imaging system (Thermo Fisher Scientific). For each of at least 3 independent experiments, every sample was represented in quadruplets, and valid objects were measured in 14 randomly selected fields.

### ATP measurements

ATP levels were measured with the CellTiter-Glo Luminescent assay (Promega), according to the manufacturer’s instructions. In brief, 5000 SUM159 cells were seeded in triplicate on a 96-well plate. At different time points, luminescence was measured after addition of the CellTiter-Glo Reagent with a SpectraMax-L luminometer (Molecular Devices, Devon, United Kingdom).

### Cell migration assay

Cells were transfected with siRNAs on a 35-mm plate and reseeded 48 h after transfection into the top chamber of a 24-well insert (pore size 8 µm; Transwell; Corning Inc., Lowell, MA, USA) at a density of 7,500, 20,000 and 30,000 for the SUM159, MCF7, and MDA-MB-453, respectively. The bottom chamber contained normal cell culture medium, and the top chamber contained medium supplemented with 1% FBS for the SUM159 or 0.5% FBS for the MCF7 and MDA-MB-453 cells. After a 24 h incubation, the cells were stained for 20 min with 0.1% crystal violet (w/v in 25% methanol). Cells on the top of the membrane were removed with a cotton swab, and the remaining cells on the bottom were imaged under a light microscope with a ×20 objective (Axiovert 100; Zeiss GmbH, Feldbach, Switzerland). Ten fields were captured per sample, and stained cells were counted using the Cell Counter plugin of ImageJ software (National Institutes of Health, Bethesda, MD, USA).

### Cell adhesion assay

Cells were seeded at a density of 25,000 per well on 96-well plates, uncoated or coated with 50 μg/ml fibronectin. After incubation for 40 min at 37°C, the plate was shaken at 600 rpm for 1.5 min. After 2 washes with PBS, the cells were fixed for 12 min with 4% formaldehyde, washed twice with PBS, and stained with crystal violet for 5 min. SDS 1% was added, and the plate was incubated with shaking at 400 rpm for 10 min, before absorbance was measured in a spectrum window of 595–600 nm with the Synergy HT microplate reader (BioTek Instruments, Winooski, VT, USA).

### Cell cycle analysis

For analysis of the cell cycle phases, cells were trypsinized and fixed in 70% ethanol for 30 min at 4°C. After treatment with 100 μg/ml RNase A treatment, staining was performed in 40 μg/ml propidium iodine dissolved in PBS. The cells were then analyzed by fluorescence-activated cell sorting (FACS) with the FACS Canto II (BD Biosciences). Data were analyzed with V9/X software (FlowJo, Ashland, OR, USA).

### Live measurements of ER lumen oxidation status

To assess the degree of oxidation in the lumen of the ER, the reduction–oxidation–sensitive green fluorescent protein (roGFP)1-iE_ER_ sensor was used (19). Cells were transfected with siRNAs and seeded on 35 mm glass-bottom dishes (MatTek, Ashland, MA, USA). After 24 h, cells were transfected with the roGFPiE_ER_ plasmid, and image analysis followed 48 or 72 h after siRNA delivery. For determination of sensor oxidation, cells were washed twice with HEPES buffer (pH 7.4) and imaged with the ×60 oil-immersion objective (NA 1.40) of a FluoView1000 confocal laser scanning microscope (Olympus, Tokyo, Japan). The 405 and 440 nm laser diodes were used to excite the sensor, and the emission window was set at 505–605 nm. The image size was 512 × 512 pixels, and the pinhole was adjusted to a completely open setting. The acquisition of the emitted fluorescence signals was sequential. Images were captured at steady state, 2 min after addition of 5 mM diamide for complete oxidation and 5 min after addition of 20 mM DTT for complete reduction of the sensor. Three independent experiments were performed, and an average of 10 cells were analyzed per sample in every experiment. In each cell 1–2 regions of interest were selected for analysis of the fluorescence emission intensities at 405 and 440 nm. Background signal was subtracted from all the acquired values. After calculation of the ratio of intensities at steady state, after complete oxidation and complete reduction, the oxidation value was calculated based on Eq. 1:
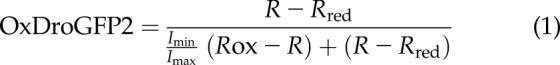
where OxDroGFP2 is the oxidation value of roGFP-2; *R*, *R*_red_, and *R*_ox_ are the 405:440 nm emission ratios at steady state or after reduction or oxidation, respectively; and *I*_min_ and *I*_max_ correspond to the fluorescence intensities of the fully oxidized and reduced sensor after excitation at 440 nm.

### Measurement of mitochondrial superoxide levels

To evaluate mitochondrial superoxide levels, we used the specific indicator MitoSox Red (Thermo Fisher Scientific). Cells were seeded on 96-well plates, and, at the desired time point, were washed with charcoal-treated medium and stained with Hoechst 33342 for 30 min at 37°C. After 1 wash with PBS and 1 wash with charcoal-treated medium, 2.5 μM MitoSox Red was added. Fluorescence intensities were measured over time with ArrayScan high-content imaging, as previously described. Because measurements were performed in living cells, the imaging chamber was humidified and maintained at an environment of 37°C and 5% CO_2_.

### Dynamic *in vivo* calcium measurements in the ER lumen

To assess ER luminal calcium content, the fluorescence resonance energy transfer (FRET)-based Cameleon sensor D1ER, which is specifically targeted to the ER, was used (20, 21). Cells were transfected with siRNAs and seeded on glass coverslips. Twenty-four hours after siRNA transfection, the cells were transfected with the plasmid containing the D1ER coding sequence. Analysis of samples commenced 42 or 68 h after siRNA transfection and, according to the time needed for sample preparation and imaging, continued up to 46 or 72 h, respectively. Cells were washed twice with 2 ml 1× HBSS (prepared from a 10× stock solution of HBSS, cat. no. 14185052; Thermo Fisher Scientific), supplemented with 20 mM HEPES buffer (pH 7.4), 2 g/L glucose, 0.49 mM MgCl_2_, 0.45 mM MgSO_4_, and 0.3 mM CaCl_2_ (henceforth referred to as HHBSS). This concentration of CaCl_2_ was based on that present in the culture medium of the SUM159 cells and had to be adjusted for each cell line. Coverslips were then transferred in a perfusion chamber, where washes and treatments were performed with the help of the P720 peristaltic pump (Instech Laboratories, Plymouth Meeting, PA, USA). The ×60 oil-immersion objective (NA 1.40) of the FluoView1000 confocal laser scanning microscope (Olympus) was used for imaging and simultaneous acquisition of emission wavelengths for cyan fluorescent protein (CFP) and FRET after excitation with the 440 nm laser diode was performed. All images were acquired in 30 s increments. Resting calcium was assessed after acquisition of 12–15 images at steady state. Subsequently, image acquisition was paused, and cells were washed 3 times with 2 ml HHBSS without CaCl_2_. Cells were then incubated in the same buffer supplemented with 10 μM ionomycin and 5 mM EGTA, and 15 more images were acquired. This treatment results in minimum concentration of calcium in the ER lumen. Data acquisition was again paused, and the cells were washed once with HHBSS without CaCl_2_ and incubated in HHBSS without CaCl_2_, supplemented with 2 mM MgCl_2_ and 100 mM ATP for 1 min. Cells were then washed once with 2 ml HHBSS (containing CaCl_2_) and incubated with HHBSS, containing an additional 5 mM CaCl_2_, 10 μM ionomycin, 2 mM MgCl_2_, and 100 mM ATP, to achieve maximum calcium concentration in the ER and thus sensor saturation. Fifteen more images were captured immediately after this treatment. The FRET ratio was calculated using Eq. 2:
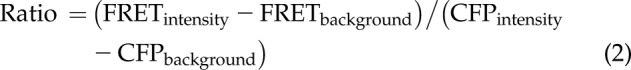
From the ratio at steady state (*R*), after ER calcium depletion (*R*_min_) and maximum ER calcium loading (*R*_max_), the Δ*R* was calculated using Eq. 3:
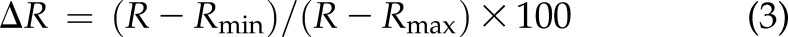
Three independent experiments were performed, and 35–40 cells per condition were imaged in total, from which 1–2 regions of interest were typically analyzed.

### Cytosolic calcium measurements

Cell were seeded and transfected with siRNAs in glass-bottom dishes. After 72 h, staining solution was added [F14201, 2.2 μg/ml FLUO-4 AM (Thermo Fisher Scientific) and 2.5 mM probenecid in complete medium], followed by incubation for 1 h at 37°C. After 3 washes with HEPES buffer, fluorescence was monitored in real time using an inverted microscope (DMI4000 B; Leica Microsystems, Buffalo Grove, IL, USA). Once the signal was stabilized, 10 mM carbachol (Cch) was added, and fluorescence was monitored until return to baseline. For fluorescence intensity measurements, an entire field visualized under the ×20 objective was chosen, and signal was quantified with Suite X software (LAS X; Leica).

### Measurements of oxygen consumption rate and extracellular acidification rate

The oxygen consumption rate (OCR) and extracellular acidification rate (ECAR) were measured in real-time using the Seahorse XF96 analyzer (Agilent Technologies). Cell line characterization was performed according to the manufacturer’s instructions, to achieve optimal cell densities and determine the concentration of carbonyl cyanide-4-(trifluoromethoxy)phenylhydrazone (FCCP) to be applied. Cells were transfected with siRNAs and seeded in 6 replicates for each sample on a 96-well microplate. On the day of the measurement, cells were washed twice with XF base medium (Agilent Technologies) and supplemented with the appropriate amounts of pyruvate, glutamine, and glucose, according to the culture medium concentrations of each cell line, and the pH was adjusted to 7.4. The cells were incubated in this medium for 1 h in an incubator without CO_2_ before transfer to the XF96 instrument. Three measurements of OCR and ECAR were performed before injection of 1 μM oligomycin and 0.5 μM FCCP, followed by 5 more measurements. During the experiment, the cells were kept in a humidified chamber at 37°C, under normal oxygen conditions. Data were processed using the Wave 2.0 software and the XF Cell Energy phenotype report generator (Agilent Technologies).

### Indirect immunofluorescence

Experiments of indirect immunofluorescence were essentially performed as in Tsachaki *et al.* (22). For actin visualization, phalloidin-FITC was used (Enzo Life Sciences, Farmingdale, NY, USA) in 1:150 dilution. The anti α-tubulin antibody was the one described in Western blot analysis and used in 1:200 dilution. All images were acquired with the ×60 objective of the FluoView 1000 confocal laser scanning microscope (Olympus).

### Statistical analysis

Data are presented as means ± sd, and the statistical significance of differences between treatments and control conditions were estimated by a 2-tailed Student’s *t* test in Prism 5 software (GraphPad, La Jolla, CA, USA). Significance was accepted at *P* < 0.05.

## RESULTS

### Knockdown of H6PD reduces proliferation and migration in the SUM159, MCF7, and MDA-MB-453 breast cancer cell lines

To examine the impact of H6PD on cancer cell proliferation, we first silenced its expression by using specific siRNAs in the SUM159, MCF7, and MDA-MB-453 cell lines. Subsequently, we performed Western blot analysis at 24, 48, and 72 h after siRNA delivery, to evaluate the efficiency of protein level reduction over time (Supplemental Fig. 1). In SUM159 cells, a reduction in H6PD levels at 24 h after siRNA treatment was not observed in all experiments, in contrast to the reduction in MCF7 and MDA-MB-453 cells. We observed discernible H6PD down-regulation at 48 h after H6PD siRNA delivery compared to mock siRNA or nontransfected controls, which was most prominent at 72 h of siRNA treatment, in all cell lines.

**Figure 1.**
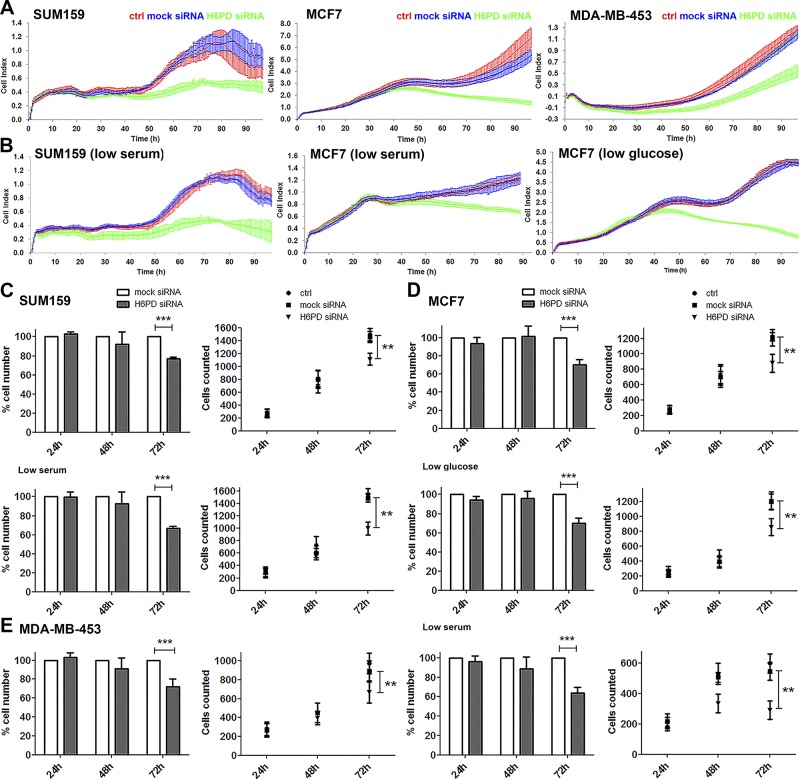
Down-regulation of H6PD reduces cell proliferation in SUM159, MCF7, and MDA-MB-453 cells. *A*, *B*) Real-time measurements of cell proliferation using a cell analyzer for SUM159, MCF7, and MDA-MB-453 cells under normal culture conditions (*A*), for SUM159 and MCF7 cells under reduced serum conditions, or for MCF7 cells under reduced glucose conditions (*B*). *C*–*E*) In each case, 1 of 3 independent measurements is shown. Red curves: untransfected control cells; blue curves: mock siRNA-treated cells; green curves: H6PD siRNA-transfected cells. Measurement of number of cells over time (24, 48, and 72 h) estimated by Hoechst nuclear staining and high-content imaging in the SUM159 cell line (normal culture and reduced serum conditions) (*C*), in MCF7 cells (normal culture, reduced serum, and reduced glucose conditions) (*D*), and in MDA-MB-453 cells (*E*). Ctrl, control (no transfection). Values represent the mean of at least 3 independent experiments; error bars = sd. *P < 0.05, **P < 0.01, ***P < 0.001 *vs.* mock siRNA.

After confirming the knockdown efficiency in our cell models, we down-regulated H6PD and monitored cell proliferation with the xCelligence RTCA. The transfection procedure did not affect proliferation, as observed when comparing nontransfected and mock siRNA-transfected cells (**Fig. 1*A, B***). In SUM159 cells, we observed a decrease in cell proliferation over time after H6PD siRNA delivery compared to mock-transfected cells, starting 48–50 h after knockdown. The same effect was observed in MCF7 cells 42–48 h and in MDA-MB-453 cells 32–40 h after knockdown (Fig. 1*A*). To investigate whether components present in the serum would further influence the effect of H6PD down-regulation on cell proliferation, we performed the same experiments in SUM159 and MCF7 cells using one-fourth of the amount of serum present in their normal medium (Fig. 1*B*; low serum). We did not observe any significant difference compared to the experiments in which normal serum concentrations were used. As stated above, it has been suggested that H6PD is involved in cellular glucose uptake (9), which would mean that a decrease in extracellular glucose concentration could deteriorate the H6PD effect on cell proliferation. Nonetheless, lowering the glucose concentration from 4.5 to 1 g/L did not cause any further reduction of MCF7 cell proliferation upon H6PD knockdown (Fig. 1*B*). The effect of lower glucose could not be tested on the other 2 cell lines, because the corresponding media were not available. To validate the above findings by a different experimental method, we performed a series of end-point experiments in which we counted nuclei after Hoechst 33342 dye staining (correlated directly with cell number) using fluorescence high-content imaging after 24, 48, and 72 h of siRNA treatment. The results are similar to those obtained with RTCA, demonstrating a considerable difference in number of cells after H6PD knockdown compared to mock-transfected cells and reaching statistical significance at 72 h after H6PD silencing for all 3 breast cancer cell lines tested (Fig. 1*C–E*). Comparison of the number of cells at 72 h after H6PD knockdown with that for mock-transfected cells at 72 h in normal culture conditions and in reduced serum for SUM159 (76.9 ± 1.9% in normal serum and 66.9 ± 1.9% in reduced serum) and MCF7 cells (70.6 ± 5.7% in normal serum and 64.1 ± 5.9% in reduced serum) or glucose concentrations for MCF7 cells (70.6 ± 5.7% in 4.5 g/L and 69.8 ± 5.4% in 1 g/L) showed no significant difference (comparison not shown).

To investigate whether the reduced number of cells upon H6PD silencing was related to defects in the cell cycle, we examined the expression of key regulatory CCNs 48 h after siRNA treatment of SUM159 cells. At this time point, cells still proliferate actively, and initial alterations in cell cycle progression can be captured. We found that the mRNA levels of CCNE1 and E2 (facilitating S transition), CCNA2 (regulating S phase and mitotic entry), and G_2_/mitotic CCNB1 were greatly reduced after H6PD down-regulation (Supplemental Fig. 2*A*). The levels of G_1_-specific CCND1 were not significantly changed, although a trend increase was observed. However, mRNA levels of the CCN-dependent kinase inhibitors p21 (gene *CDKN1A*) and p27 (gene *CDKN1B*), which control progression of the cell cycle at the G_1_ checkpoint, were increased. In accordance, cell cycle analysis at 48 h after H6PD down-regulation, showed a higher percentage of cells at the G_1_ phase (Supplemental Fig. 2*B*). Increased accumulation of cells at this stage could eventually lead to a reduced number of cells after H6PD silencing.

**Figure 2.**
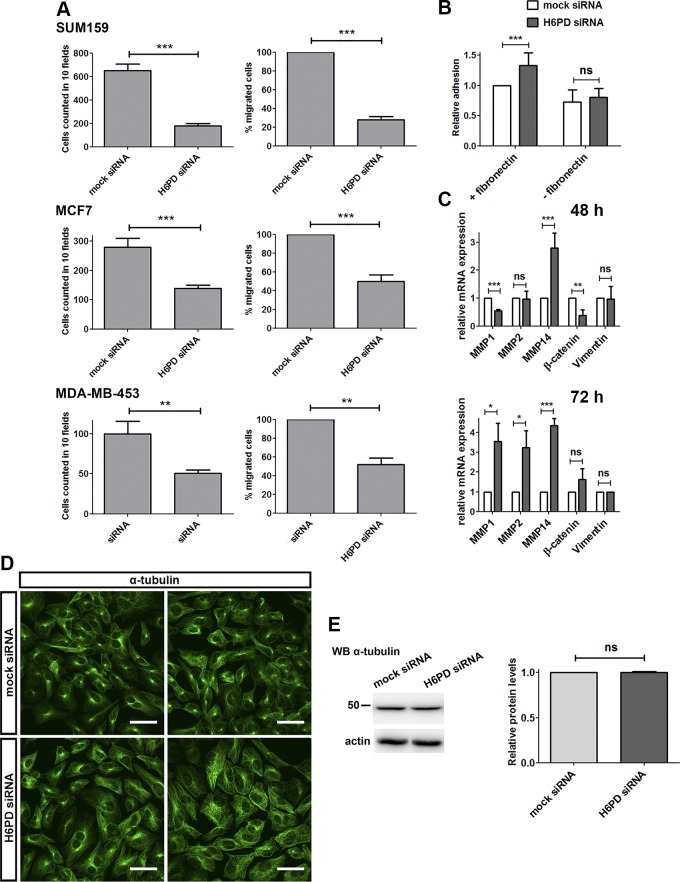
H6PD knockdown reduces cancer cell migration and increases cell adhesion. *A*) SUM159, MCF7, and MDA-MB-453 cells were transfected with mock or H6PD siRNAs, and cell migration was assessed by a migration assay. Data represent the number of migrated cells counted after crystal violet staining in 10 independent visual fields (left) or as a percentage of migrated cells after H6PD knockdown, compared with mock-transfected cells (right). Graphs depict the means from at least 3 independent experiments ± sd in SUM159 cells. mRNA analysis of the indicated genes 48 h after H6PD knockdown in SUM159 cells. *B*) SUM159 cells were transfected with mock or H6PD siRNAs and reseeded after 72 h on fibronectin-coated plates or were left untreated. Adhesion was evaluated spectrometrically after crystal violet staining. Absorbance data were normalized to the values of mock-transfected cells on fibronectin-coated wells. *C*) qPCR analysis of mRNA levels for the depicted genes, 48 or 72 h after siRNA transfection in SUM159 cells. *D*) SUM159 cells were transfected with H6PD siRNA or mock siRNA and, 72 h later, were subjected to immunofluorescence with an antibody against α-tubulin. *E*) Western blot analysis of α-tubulin levels in SUM159 cells 72 h after knockdown of H6PD compared to mock-transfected cells. Ns, nonsignificant. *P < 0.05, **P < 0.01, ***P < 0.001 *vs.* mock siRNA.

Besides increased proliferation potential, a central aspect of tumor cell physiology is their ability to migrate and invade distant tissues. To evaluate whether H6PD is involved in cancer cell migration, we performed a cell migration assay (Transwell) with a serum gradient as a chemoattractant. H6PD knockdown led to an 80% decrease in migration of SUM159 cells, which had the highest migratory potential of the 3 cell lines analyzed (**Fig. 2*A***). A significant decrease in cell migration was also observed in the MCF7 and MDA-MB-453 cells.

**Figure 3.**
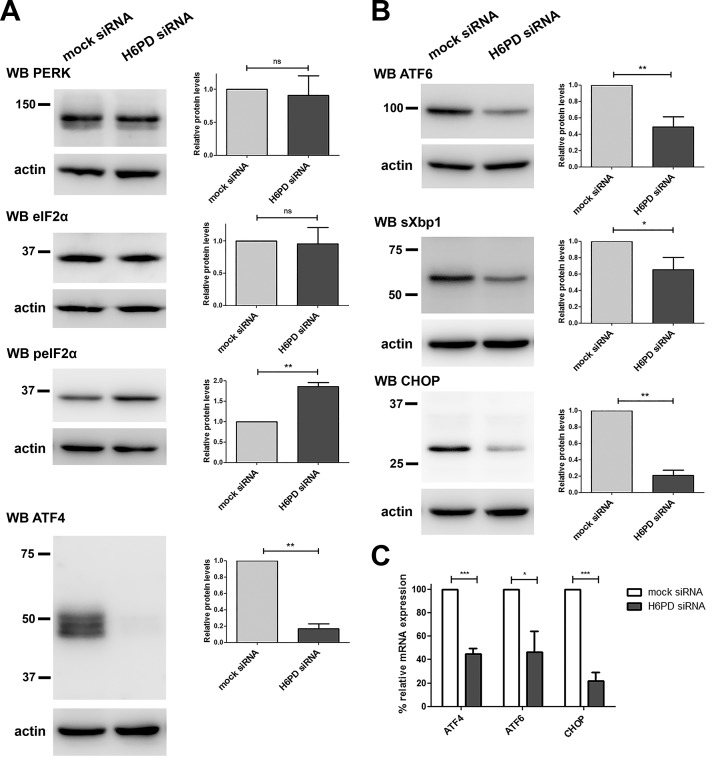
Alterations in UPR signaling after H6PD silencing. SUM159 cells were mock or H6PD siRNA transfected. *A*, *B*) Western blot analysis was performed at 72 h after transfection for semiquantitative analysis of protein levels of PERK, eIF2α, peIF2α, and ATF4 (*A*) and ATF6, sXBP1, and CHOP (*B*). A representative Western blot image is shown (left) in addition to the relative protein levels measured from 3 independent experiments by densitometric analysis of the bands after normalization to actin loading control levels (right). *C*) mRNA expression of ATF4, ATF6, and CHOP 72 h after H6PD down-regulation compared to mock-transfected cells, as assessed through qPCR. Values were normalized to the levels of the gene prolyl *cis-trans* isomerase A. Ns, nonsignificant. *P < 0.05, **P < 0.01, ***P < 0.001 *vs.* mock siRNA.

**Figure 4.**
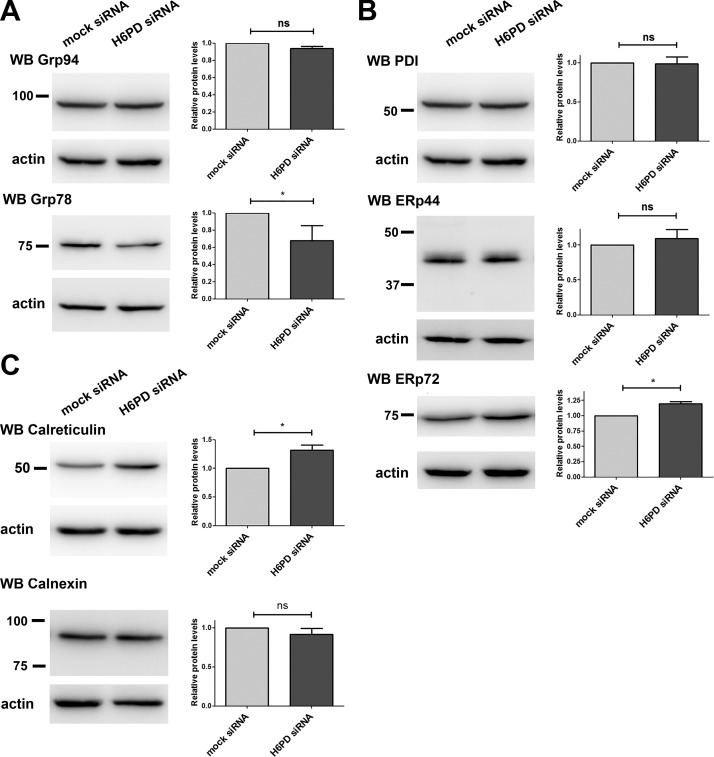
Effect of H6PD knockdown on ER folding proteins. H6PD or mock-transfected SUM159 cells were analyzed by Western blot, as in Fig. 3, for estimation of levels of the heat shock family proteins Grp94 and -78 (*A*), the PDI family proteins PDI, ERp44, and ERp72 (*B*) and the chaperones calreticulin and calnexin (*C*). Ns, nonsignificant. *P < 0.05 *vs*. mock siRNA.

Altered migration may be related to changes in cell adhesion. To explore this possibility, we studied the impact of H6PD on the attachment to fibronectin-coated dishes of SUM159 cells and found a significant increase in adhesion upon H6PD silencing (Fig. 2*B*). To examine whether this effect derives from altered expression of proteins responsible for cell interaction with the extracellular matrix, we examined the expression of matrix metalloproteinases (MMPs), focusing on the MMPs that were highly expressed in our cell model. Forty-eight hours after transfection of H6PD siRNAs in SUM159 cells, the levels of MMP1 were decreased, consistent with the observed increase in cell adhesion (Fig. 2*C*). MMP1 is transcriptionally activated by β-catenin (gene name *CTNNB1*), which was also found to be down-regulated (23). Reduced β-catenin levels could lead to attenuated expression of other genes that promote cell migration, as well as changes in the integrity of the cytoskeleton (24). In addition, MMP2 levels remained the same, whereas the levels of MMP14 were surprisingly increased. In fact, at 72 h after H6PD down-regulation, all MMPs tested were up-regulated, which could be explained through the existence of a compensatory mechanism. Next, we sought to determine whether defects in cytoskeletal organization account for reduced migration. For this purpose, we examined the pattern of actin filaments as visualized after binding to phalloidin-FITC, and encountered no difference between mock and H6PD siRNA-transfected SUM159 cells (Supplemental Fig. 3*A*). Examination of the microtubule scaffold through α-tubulin staining showed unchanged overall organization (Fig. 2*D*). However, in the case of H6PD silencing, the microtubule organization center (or centrosome), from where the fibers emanate, exhibited a more diffuse pattern. The expression level of neither actin (**Figs. 3** and **4**) nor α-tubulin (Fig. 2*E*) was affected by H6PD knockdown. Finally, analysis of mRNA levels of the intermediate filament protein vimentin showed no difference after H6PD silencing (Fig. 2*C*).

### Decreased expression of proteins involved in the UPR pathway upon H6PD down-regulation

In muscle cells from H6PD^−/−^ mice, changes in proteins of the UPR pathway have been reported (7). Emerging evidence strongly suggests that mild activation of the UPR pathway is essential for cancer cell growth (25, 26). Therefore, we investigated whether H6PD could affect cancer cell proliferation through changes in the UPR. This pathway consists of 3 branches, and extensive crosstalk between components of those branches have been documented (reviewed in ref. 27). The first branch commences with the PERK, which, upon activation through autophosphorylation, promotes phosphorylation and activation of eIF2α. This factor then acts as a global inhibitor of mRNA translation, but specifically increases translation of certain mRNAs, predominantly that of ATF-4, which subsequently enhances transcription of target genes, including CHOP.

To investigate the possible involvement of H6PD in this pathway, we down-regulated its expression in SUM159 cells and performed Western blot analysis against PERK, eIF2α, peIF2α, and ATF4 at 72 h after siRNA transfection (Fig. 3*A*). We did not detect significant changes in PERK expression levels upon H6PD knockdown. However, we could not find an antibody against the phosphorylated form of PERK that worked in our hands, and therefore we could not draw conclusions on its activation status. Although total eIF2α levels were unaffected, a significant increase in its phosphorylated form was observed after H6PD knockdown. Although one would expect this to result in increased ATF4 levels, we paradoxically observed a dramatic decrease in ATF4 expression, which could imply that higher eIF2α phosphorylation represents a compensatory adaptation. Decreased ATF4 levels upon H6PD silencing were also observed at the mRNA expression level (Fig. 3*C*).

The second branch of the UPR response includes inositol-requiring (IRE)-1α, which, upon activation and phosphorylation, leads to the splicing of the sXBP1, producing the active transcription factor sXBP1, that upon translocation to the nucleus, leads to the expression of target genes, often involved in ERAD (28, 29). Our results showed significantly decreased sXBP1 levels upon H6PD knockdown (Fig. 3*B*). The third branch of the UPR includes ATF-6, an ER membrane protein that upon activation translocates to the Golgi where it is proteolytically cleaved. The active transcription factor fragment of the protein is then released and controls target gene expression in the nucleus. Upon H6PD knockdown, we observed a significant decrease in ATF6 expression, at both the transcriptional (Fig. 3*C*) and protein level. In line with the above findings, the mRNA and protein levels of CHOP, with expression that can be induced by both ATF4 and -6, were significantly decreased (Fig. 3*B, C*). These results point toward an attenuation of all branches of the UPR upon H6PD silencing. To test whether the observed differences are caused by the decreased cell proliferation, we performed the same experiments 42 h after H6PD knockdown, at a time point before the proliferation of the SUM159 cells was affected. We obtained identical results for ATF4 and CHOP at 72 h, suggesting that the reduction in the expression levels of these 2 transcription factors precedes the decrease in cell proliferation (Supplemental Fig. 4). ATF6 expression tended to decrease 42 h after H6PD knockdown, whereas sXBP1 levels were unchanged.

### Effect of H6PD knockdown on the protein-folding machinery

The UPR response can be elicited by a variety of stimuli, including defects in protein folding or protein-folding overload in the ER. To investigate whether the observed differences after H6PD down-regulation in the UPR protein levels are a consequence of changes in protein folding, we analyzed the expression of members from the 3 main protein-folding families (30, 31). First, we examined the heat-shock proteins Grp94 (the ER paralogue of cytosolic heat shock protein 90) and Grp78. We found no difference in the levels of Grp94 after H6PD silencing, compared with mock-transfected SUM159 cells, whereas Grp78 expression was slightly reduced (Fig. 4*A*). Next, we tested the expression of members of the PDI family. H6PD knockdown caused no change in the expression of PDI and ERp44, but there was a small but significant increase in ERp72 levels (Fig. 4*B*). Finally, we tested the expression of calreticulin and calnexin, which serve as molecular chaperones for glycoproteins in the ER. H6PD down-regulation caused a significant increase in calreticulin levels, whereas calnexin expression was unchanged (Fig. 4*C*).

### H6PD silencing alters SERCA2 and IP3RIII expression and increases intraluminal Ca^2+^

**Figure 5.**
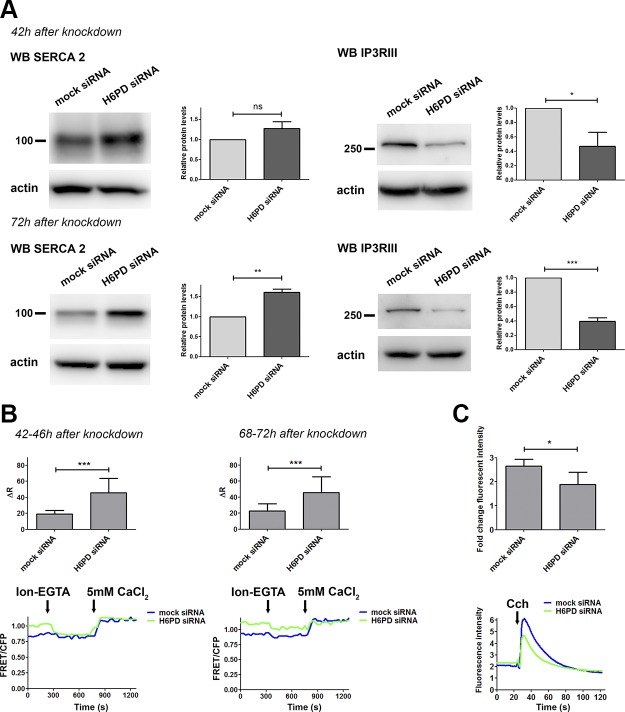
H6PD knockdown alters the expression of ER calcium transporters and intraluminal calcium concentrations. *A*) The expression of SERCA2 and IP3RIII was analyzed by Western blot 42 and 72 h after siRNA treatment in SUM159 cells. *B*) ER luminal calcium concentrations were measured 42–46 and 68–72 h after H6PD knockdown by using the D1ER probe. In total, 35–40 single cells were imaged per condition in 3 independent experiments. The graphs depict the mean ± sd of Δ*R* values after baseline measurements, followed by calibration of the sensor with ionomycin (Ion)/EGTA for *R*_min_ determination, and subsequent addition of Ion/5 mM CaCl_2_ for *R*_max_ determination. A representative plot of the FRET ratio (FRET:CFP) is shown at the bottom of each graph. *C*) At 72 h after transfection with the indicated siRNAs, SUM159 cells were incubated with FLUO-4 AM. Fluorescence was measured in real-time at steady state and after addition of Cch. At the top of the panel the normalized values of the difference in fluorescence intensity before and after Cch treatment are depicted, and at the bottom, the actual measurement during a representative experiment. Ns, nonsignificant. *P < 0.05, **P < 0.01, ***P < 0.001 *vs.* mock siRNA.

The 2 calcium-binding proteins calreticulin and ERp72 were the only ones of all the ER chaperones examined that exhibited a significant increase in their expression after H6PD knockdown. This result led us to test a potential effect of H6PD down-regulation on proteins responsible for maintaining ER calcium homeostasis. H6PD silencing in SUM159 cells caused a significant increase in SERCA2 expression, which mediates calcium transport from the cytosol into the ER (**Fig. 5*A***). This increase was observed both after 42 h, before a significant difference in cell proliferation could be detected, and after 72 h of siRNA treatment, but reached statistically significant levels only after 72 h. Furthermore, the levels of the IP3RIII, which is responsible for calcium export from the ER, were significantly reduced both 42 and 72 h after H6PD knockdown (Fig. 5*A*). We reasoned that the increased SERCA2 levels, together with decreased IP3RIII expression upon H6PD silencing, may be caused by an increase in ER calcium concentrations. Alternatively, H6PD knockdown could lead to an ER-to-cytoplasm calcium leak, which may be compensated for by the observed changes in SERCA2 and IP3RIII expression. To discriminate between the 2 potential mechanisms, we monitored ER luminal calcium levels by using the FRET-based indicator D1ER (21). This sensor enables live measurements of calcium in individual cells. The critical point in using fluorescent calcium indicators is the calibration of the sensor for each cell measured. Therefore, a calibration protocol has to be developed for every cell line to determine the minimum and maximum calcium-binding capacity of the sensor. For the SUM159 cell line, to reach the minimum FRET ratio (FRET:CFP), 10 μM ionomycin and 5 mM EGTA in calcium-free buffer were used, whereas to reach maximum FRET ratio, 10 μM ionomycin and 5 mM CaCl_2_ were used (for details, see Materials and Methods). We observed a significant increase in FRET efficiency both at 42 and 72 h after H6PD knockdown, which translates into increased ER luminal calcium concentrations, starting at least 42 h after siRNA treatment (Fig. 5*B*). To examine whether this increase could be related to reduced calcium release from the ER, we treated cells with the cytosolic calcium indicator FLUO-4 AM (Thermo Fisher Scientific) and monitored fluorescence in steady state and after addition of Cch, which induced release from ER calcium stores through IP3 receptors. From these experiments, we cannot report absolute cytosolic calcium levels, because no calibration of the system was performed. We observed that, after H6PD down-regulation, there was a significant decrease in the difference between fluorescence intensity at baseline and after Cch addition (Fig. 5*C*), suggesting defective calcium efflux from the ER.

### Down-regulation of H6PD leads to an increased oxidation state in the ER lumen and superoxide production in mitochondria

**Figure 6.**
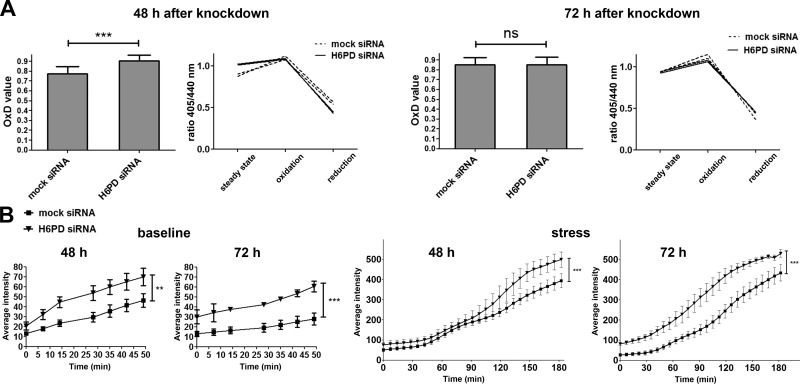
Silencing of H6PD leads to an increase in the ER redox status and mitochondrial superoxide levels. *A*) SUM159 cells were transfected with mock or H6PD siRNAs, and redox changes were observed 48 and 72 h after treatment, using the ER-targeted ratiometric probe roGFP1-iE_ER_. The OxD value was determined from the ratios of fluorescence emission intensities after excitation at 405 and 488 nm. Means ± sd were calculated from 3 independent experiments. Representative graphs of the ratio of fluorescence intensities at baseline, after full oxidation and full reduction of the sensor are shown from 2 H6PD- and 2 mock-siRNA–transfected cells at each time point. *B*) H6PD was down-regulated in SUM159 cells, and 48 or 72 h later, superoxide levels were monitored using the red indicator under normal conditions or after addition of ethanol as stressor. The measurements were performed in real time by using high-throughput imaging, and the values represent the mean of 3 independent experiments. Baseline and stressed levels were documented in each experiment and are shown separately for better comprehension of scale. For simplicity, only the statistical significance of the latest time point is depicted in every graph. Ns, nonsignificant. **P < 0.01, ***P < 0.001 *vs.* mock siRNA.

It is known that the environment in the ER is more oxidized than in the cytosol, thereby supporting oxidative protein folding (19, 32). The redox conditions in the ER and the UPR machinery are unambiguously linked (33). Furthermore, recent findings suggest that ER calcium signaling proteins are also affected by the ER redox status (34). Because H6PD knockdown leads to decreased NADPH content in the ER lumen, we investigated whether this would cause a change in the ER redox environment that could offer a link to the observed UPR and calcium changes. To assess the ER oxidation status, we used an ER-targeted roGFP, roGFP1iE_ER_. The challenge with roGFP sensors targeted to the ER lumen is that they exhibit a high degree of oxidation at steady levels, because of the highly oxidized environment in the ER lumen. This renders measurement of changes toward an increase of oxidation particularly challenging. The choice of roGFP1-iE_ER_ was based on the fact that it was the least oxidized ER-specific roGFP sensor among a series of constructs reported earlier (19). The ratio of fluorescence intensity emission after excitation at 405 and 440 nm at steady state, after complete oxidation (diamide addition) and complete reduction (DTT addition) of the sensor, enables the calculation of the OxD value, representing the degree of oxidation. Live-imaging experiments were performed starting 48 or 72 h after mock- or H6PD-siRNA transfection in SUM159 cells, and the OxD values were calculated after measurements of fluorescent emission intensities in individual cells. At 48 h after knockdown, we observed a significant increase in oxidation upon H6PD silencing, which was no longer evident at 72 h. This finding is depicted by both the fluorescence ratio in the 3 oxidation states of the sensor and the OxD values (**Fig. 6*A***).

Next, we asked whether the increase in ER oxidation would lead to augmented production of reactive oxygen species (ROS). Unfortunately, we were not able to successfully implement the ER-specific hyper probe in our cell system (35). Given the physical and functional connection between the ER and mitochondria, we next tested whether ROS content in mitochondria could be affected by H6PD down-regulation. For this purpose, we used the mitochondrial superoxide anion indicator MitoSOX Red. We found that, at both 48 and 72 h after knockdown, baseline levels of superoxide were higher in the H6PD siRNA-transfected cells (Fig. 6*B*). This difference increased over time under the constant presence of a stressor that augments mitochondrial superoxide production (15% ethanol). The above findings imply that silencing of H6PD expression causes elevated ROS production in mitochondria.

### Impact of H6PD knockdown on energy metabolism

**Figure 7.**
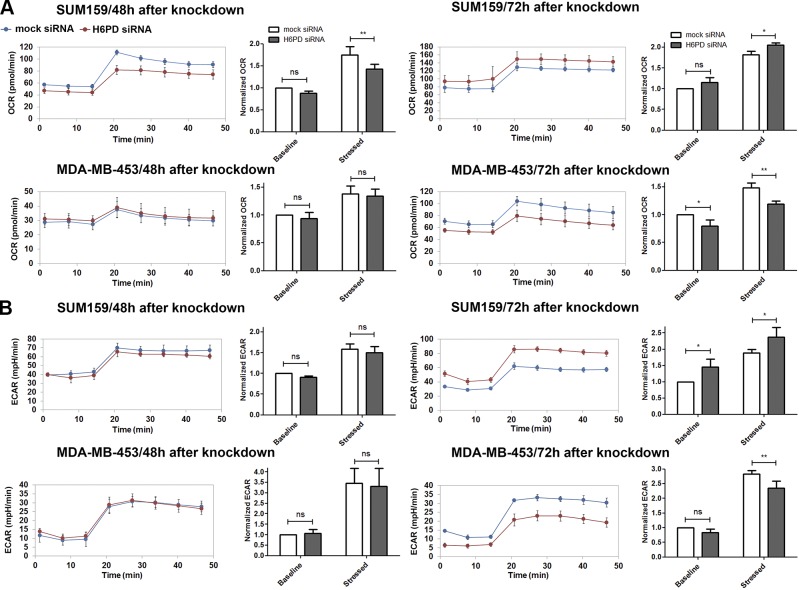
Changes in energy metabolism after H6PD knockdown. OCR (*A*) and ECAR (*B*) were measured 48 and 72 h after H6PD knockdown in SUM159 and MDA-MB-453 cells. Three baseline measurements were performed followed by treatment with oligomycin/FCCP and 5 additional measurements. In each case, a representative real-time measurement is shown (left), in addition to the values of OCR and ECAR from 3 independent experiments normalized to the baseline levels of mock-transfected cells (right). Ns, nonsignificant. *P < 0.05, **P < 0.01 *vs.* mock siRNA.

Previous work showed that H6PD down-regulation in 2 murine cancer cell lines decreased overall ATP levels, without affecting mitochondrial oxygen consumption. In addition, supernatant lactate concentration, indicative of glycolysis, was found to be decreased (9). In the present study, measurement of total ATP levels in correlation with the total number of cells in the SUM159 cell line, did not show statistically significant changes between mock- and H6PD siRNA–treated cells at 24, 48, and 72 h after transfection, although the levels tended to be higher for the cells where H6PD was down-regulated (Supplemental Fig. 3*B*). To assess specific aspects of energy metabolism in the 3 selected human breast cancer cell lines, we performed real-time measurements of OCR (indicative of mitochondrial respiration) and ECAR at steady state levels and after application of antimycin and FCCP according to the Seahorse manufacturer’s protocol (Agilent Technologies). Antimycin inhibits the electron transport chain in mitochondria, provoking a concomitant increase in glycolysis. FCCP is an uncoupler of oxidative phosphorylation in mitochondria and drives a compensatory increase in oxygen consumption. Combination of both substances is expected to raise both OCR and ECAR levels, allowing for estimation not only of the steady state of cells, but also of their metabolic potential after stress. It should be noted that OCR is a good indicator of oxidative phosphorylation, but enzymatic oxygen consumption not related to mitochondrial respiration could account for a small percentage of our measurements and cannot be discriminated with the assay used. We found that H6PD knockdown in SUM159 cells led to decreased OCR 48 h after transfection, which was more pronounced after stress (**Fig. 7*A***). This effect was reversed 72 h after knockdown, where a rise in OCR levels was observed after H6PD knockdown, indicating the presence of a compensatory mechanism. On the other hand, MDA-MB-453 cells exhibited a decreased OCR, both at baseline and stressed levels, only 72 h after H6PD silencing. ECAR levels were not significantly affected in SUM159 cells 48 h after H6PD down-regulation, whereas an increase was observed after 72 h (Fig. 7*B*). In contrast, reduced ECAR, which was statistically significant only after stress, was detected in MDA-MB-453 cells. Although glycolysis is the main reason for medium acidification, other sources may contribute to the extracellular proton concentration. To confirm that the ECAR measurements mirror changes in glycolysis in our cell systems, we studied at steady state levels the expression of 2 conventional markers of glycolysis, hexokinase-1, and ATP-dependent 6-phosphofructokinase, liver type, (Supplemental Fig. 3*C*). We showed that, at 48 h after H6PD silencing in the SUM159 cells, the levels of both enzymes were unaltered, whereas an increased expression was observed at 72 h. These results are consistent with the baseline ECAR measurements for this cell line at the 2 time points (Fig. 7*B*).

## DISCUSSION

The cytosolic pentose phosphate pathway produces reducing equivalents in the form of NADPH, as well as pentoses, including the nucleotide precursor ribose 5-phosphate. The first step in this metabolic cascade is the conversion of glucose 6-phosphate to 6-phosphogluconolactone, with concomitant NADPH production, and it is performed by G6PD. The ER equivalent of G6PD is H6PD, and it is the only well-characterized enzyme known so far to produce NADPH within the ER. A latent 6-phosphogluconate dehydrogenase activity generating NADPH in the ER lumen has been observed in isolated rat liver microsomes [(36) and Legeza and Odermatt, unpublished observations]; however, the gene encoding this enzyme has not yet been identified. In addition, although evidence of the existence of other downstream components of the pentose phosphate pathway in the ER has been provided (37), the genes corresponding to the observed enzymatic activities remain to be discovered. Therefore, whether the ER contributes to total cellular ribose production through an independent pentose phosphate pathway, thereby affecting ATP production, remains unclear. Establishment of a reliable method for measurement of ribose levels in the ER could partially address this central question. Because of poor membrane permeability, the ER pool of NADPH is maintained independent of that in the cytosol. To date, 11β-HSD1 represents the only enzyme that has undeniably been shown to use NADPH as a cofactor in the ER and to perform a reductive reaction. 11β-HSD1, known to catalyze the oxoreduction of cortisone as well as that of 7-ketocholesterol and 7-ketolithocholic acid (38), is expressed in only 1 of the 3 cell lines investigated in the present study (SUM159) and thus cannot explain the effects of H6PD knockdown on the proliferation and migratory potential of the 3 different breast cancer cell lines. This finding renders the importance of luminal NADPH production by H6PD enigmatic. In the current study, we showed that H6PD supports proliferation in 3 different breast cancer cell lines. Whether the impact of H6PD knockdown on proliferation is caused by lack of luminal NADPH or impaired formation of products of the ER luminal pentose phosphate pathway is not known. Forty-eight hours after H6PD knockdown, key CCNs for cell cycle progression exhibited reduced expression, and a higher percentage of cells were at the G_1_ phase. G_1_ block after H6PD down-regulation has been reported before, albeit with a more pronounced difference (9). In the latter study, the time point after knockdown was not reported, and at the time of the experiment, most of the cells were in the G_1_ phase. These parameters could affect the magnitude of the observed effect.

Furthermore, we found that silencing of H6PD causes a dramatic decrease in cancer cell migration. Reduced migration was accompanied by an increase in cell adhesion. The mRNA levels of MMP1 and β-catenin (which transcriptionally regulates MMP1) (23) were decreased 48 h after H6PD knockdown, consistent with stronger adhesion of cells to the extracellular substrate. However, all 3 MMPs tested (MMP1, -2, and 14) were up-regulated at the mRNA level 72 h after H6PD down-regulation. This finding could be attributable to an attempt of the cells to balance increased adhesion after H6PD knockdown. In this case, the reduction of β-catenin could be the primary effect. MMPs are known to be regulated at multiple levels, including proteolysis of precursor proteins (pro-MMPs) and regulation of activity through tissue inhibitors of metalloproteinases [reviewed in (39)]. Therefore, elevated MMP mRNA levels could alternatively be a compensatory mechanism for reduced pro-MMP cleavage or MMP activity. Upon H6PD silencing, we observed a loose appearance of the centrosome, as visualized through α-tubulin immunofluorescence staining. Microtubule structure is indispensable for cell movement (40), and changes in the centrosome have been reported to cause defects in cell migration (41, 42). More detailed studies on the structure and composition of the microtubular network after H6PD down-regulation will provide a clearer picture.

Mice lacking H6PD present with skeletal muscle defects and muscles cells from these animals exhibited increased Grp78 protein expression, in addition to alterations in different UPR proteins, as shown by microarrays, including an induction of CHOP (7). To characterize the role of H6PD in cancer cell proliferation and migration, we analyzed the cellular changes after H6PD silencing. A detailed analysis in our SUM159 breast cancer cell system of key proteins involved in the 3 different UPR branches showed decreased expression of the chaperone Grp78 and the transcriptions factors ATF4, ATF6, sXBP1, and CHOP. The reduction in ATF4 and CHOP expression preceded the decreased proliferation, indicating that these factors act upstream of the latter event. Those findings point toward a role of H6PD in pathways activating ATF4, which most typically involve oxidative damage and amino acid depletion (43). Using the roGFP1iE-ER sensor in live single-cell measurements, we demonstrated that H6PD knockdown provoked an increased oxidation state in the ER lumen, which was present 48 h after knockdown and returned to basal levels after 72 h, suggesting the ability of the cells to reverse this imbalance. H6PD down-regulation also caused an increase in mitochondrial superoxide levels, reiterating the notion that changes in ER homeostasis affect mitochondrial function (44, 45).

Because there is extensive crosstalk among the 3 UPR pathways, it is critical to clarify which are the initial UPR components affected: ATF4 promotes *CHOP* and *XBP1* expression (46, 47), and ATF6 induces transcription of *XBP1* (48). Nonetheless, the 3 UPR branches are responsive to different stimuli and dedicated to regulation of diverse cellular functions. The specific molecules and pathways affected by the reduction in the levels of ATF4, ATF6, CHOP, and sXBP1 after H6PD down-regulation necessitate further analysis. Among several interesting candidates are autophagy-related genes controlled by ATF4 and CHOP (such as, *ATG5*, *ATG12*, and *P62*) (49, 50) and ERAD-associated genes regulated by XBP1, ATF6, or both, including ER degradation-enhancing α-mannosidase-like protein and DnaJ homologue subfamily B member 9 (51, 52). Furthermore, the metastasis-promoting factors VEGFA and lysosomal-associated membrane protein-3) are activated by ATF4 and are likely to be affected after H6PD knockdown (53, 54).

The reliance of cancer cells on the UPR pathway has been well established, and increased levels of critical UPR components have been shown in a multitude of human cancer tissues, including those originating in breast, colon, lung, pancreas, skin, prostate, and brain (25, 26, 55, 56). It is conceivable that constitutive UPR activation is important for excessive cell proliferation, which is coupled to high rates of protein synthesis and demand for protein-folding regulation. Moreover, cancer cells are often forced to survive in conditions of hypoxia and nutrient depletion, which drives them to harness the UPR system for counterbalancing these harsh conditions. Therefore, the UPR could well represent the Achilles’ heel in malignant cell growth, and therapeutic strategies to target this pathway are currently being explored (57–59). This strategy involves both attempts to overactivate and inhibit the UPR. For example, several compounds targeting either the ATP-binding pocket or the RNase domain of IRE1, and thereby reducing XBP1 mRNA cleavage, have been developed, and encouraging results in preclinical studies for multiple myeloma have been reported (60, 61). The PERK-eIF2α-ATF4 pathway has also attracted considerable attention, with recently developed PERK inhibitors showing promising antitumor effects in mouse models (62, 63). In light of the above, identification of H6PD as a modulator of UPR in cancer cells is of particular interest and offers a potential therapeutic target, especially in view of the mild phenotype reported in mice lacking H6PD.

In an attempt to elucidate the mechanism underlying the attenuation of UPR after H6PD silencing, we examined a potential involvement of impaired protein folding. We found significantly increased levels of calreticulin, a calcium-binding protein that surveils proper folding of glycoproteins in the ER lumen before exit to the Golgi (64). In addition, the levels of ERp72, a PDI family member that catalyzes disulfide bond formation and contains a calcium-binding domain, were increased (65). Because of the changes in the 2 calcium-binding chaperones, we investigated the impact of H6PD on ER calcium homeostasis and found increased IP3RIII expression after 42 h of H6PD knockdown, a time point preceding the reduced cell proliferation. SERCA2 expression was significantly up-regulated after 72 h of H6PD down-regulation. IP3RIII is responsible for calcium export from the ER, whereas SERCA2 imports calcium from the cytosol into the ER. Reduced SERCA2 expression has been reported in many types of human cancer, including oral and thyroid cancer (66, 67), which raises the possibility that the ER calcium content in cancer cells at steady state is lower than that of normal cells. Live measurements of calcium in the lumen of the ER using the D1ER FRET-based sensor indicated increased calcium levels after H6PD knockdown, beginning at 42 h after knockdown and persisting at least until 72 h. Although this increase occurred soon after H6PD down-regulation, further experiments are needed to learn whether this is a primary effect caused by reduced H6PD expression or a compensation for a presumptive calcium leak. Our experiments revealed attenuated ability of calcium release from the ER upon Cch addition after H6PD down-regulation. Whether the changes in calcium have a causative role in the observed UPR alterations or are a consequence of those remains to be determined. Deviations from normal calcium concentrations in the ER lumen are likely to influence both cancer cell proliferation and migration, because calcium signaling has been shown to play a paramount role in cancer cell survival (68).

According to a previous study, knockdown ofH6PDin 2 murine cancer cell lines caused a reduction in ATP content, which was attributed to a reduction in glucose consumption, whereas no difference in oxidative phosphorylationwas observed (9). In the present study, in analyzing SUM159 cells, we did not find differences in totalATP levels afterH6PDdown-regulation.As different cancer cells can display profound differences in their metabolic capacity and in using different energy sources, we determinedthe impact ofH6PDknockdown on energy metabolism in the 2 breast cancer cell lines SUM159 and MDA-MB-453, both in their basal state and under stressed conditions by treatment with the mitochondrial electron transport chain inhibitor antimycin and the oxidative phosphorylation uncoupler FCCP. Comparison of the baseline and stressed phenotype provides insight into the metabolic potential of each cell line. Knockdown of H6PD led to increased ECAR in the SUM159 cells and decreased OCR in the MDA-MB-453 cells at steady state. Analysis of the rate of OCR/ECAR utilization (Supplemental Fig. 3*D*) showed that the SUM159 cells equally depend on both sources, whereas the MDA-MB-453 cell line mostly uses oxidative phosphorylation in comparison to glycolysis for energy production. Our results indicate that a main energy source is affected in both cases. The increased ECAR in SUM159 cells 72 h after H6PD knockdown, which could be caused by a compensatory capacity of this cell line, makes interpretation of ECAR results difficult, warranting further investigation. Because the MDA-MB-453 cells did not show differences in basal levels of glycolysis after H6PD knockdown, our study is inconclusive as to whether H6PD is generally involved in glucose metabolism. After stress, the SUM159 cells also exhibited a reduced OCR 48 h after H6PD down-regulation, which was observed in the MDA-MB-453 cells after 72 h under normal and stressed conditions. This finding suggests that the SUM159 cell line is resistant to changes in OCR, and differences are seen only after stress. Moreover, the SUM159 cells exhibit significantly increased oxygen consumption only after stress 72 h after H6PD silencing, which could represent a compensatory mechanism related to defects in oxidative phosphorylation. The MDA-MB-453 cell line demonstrates a great metabolic glycolytic potential after stress, which was shown to be reduced after H6PD knockdown. Our findings point toward an unanticipated change in energy metabolism after H6PD down-regulation. It is possible that the effects of H6PD on energy metabolism, especially regarding OCR, are linked with the altered ER redox environment, or the increased ROS production in mitochondria (Fig. 6). In SUM159 cells after 48 h of H6PD knockdown, before possible compensatory adaptations, OCR decreased upon treatment with the stressors antimycin and FCCP, whereas superoxide production increased both under basal conditions and after exposures to ethanol as a stressor. This effect suggests a real reduction in oxidative phosphorylation activity, greater than that indicated by the OCR measurement. Given the flexibility in energy source utilization by cancer cells, further investigation into this topic requires extensive understanding of energy metabolism in each cell system. It needs to be noted, that the metabolic pathways of different breast cancer cell lines might be profoundly influenced by the culture conditions and medium composition, which may contribute to the differences in OCR and ECAR observed in the present study and that they may differ from those in patient’s breast tumor cells.

The results presented highlight the importance of NADPH production in the ER through H6PD for cancer cells. We demonstrated a significant role for H6PD in promoting cell proliferation and migration. Moreover, we described the cellular changes after H6PD silencing that could underpin these effects, including an increased ER luminal oxidation state within the ER, an attenuation of the UPR pathway and an increase in ER calcium concentrations. The ER is a dynamic organelle where vital functions take place, encompassing, among others, protein synthesis and quality control, lipid synthesis, and calcium signaling. Our study opens new avenues into understanding how compromised ER homeostasis influences cancer cell physiology and proposes H6PD as a potential novel therapeutic target against cancer.

## Supplementary Material

This article includes supplemental data. Please visit *http://www.fasebj.org* to obtain this information.

## Abbreviations

11β-HSD1: 11β-hydroxysteroid dehydrogenase type 1
ATF: activating transcription factor
Cch: carbachol
CCN: cyclin or G_1_/S-specific cyclin
CFP: cyan fluorescent protein
CHOP: CCAAT/enhancer binding protein homologous protein
ECAR: extracellular acidification rate
eIF2α: eukaryotic initiation factor 2α
ER: endoplasmic reticulum
ERAD: ER-associated degradation
FACS: fluorescence-activated cell sorting
FBS: fetal bovine serum
FCCP: carbonyl cyanide-4-(trifluoromethoxy)phenylhydrazone
FRET: fluorescence resonance energy transfer
G6PD: glucose 6-phosphate dehydrogenase
Grp: glucose-regulated protein
H6PD: hexose-6-phosphate dehydrogenase
HEPES: hydroxyethyl piperazineethanesulfonic acid
Her: human epidermal growth factor receptor
HHBSS: HEPES-HBSS
IP3R: inositol trisphosphate receptor
MMP: matrix metalloproteinase
OCR: oxygen consumption rate
OxD: oxidation
PARP: poly(ADP-ribose) polymerase
PDI: protein disulfide isomerase
PERK: protein kinase R-like ER kinase
qPCR: quantitative PCR
roGFP: reduction–oxidation-sensitive green fluorescent protein
ROS: reactive oxygen species
RTCA: real-time cell analyzer
SERCA: sarco/endoplasmic reticulum Ca^2+^-ATPase
siRNA: short interfering RNA
sXBP1: split X-box binding protein 1
UPR: unfolded protein response
