# Supplementary figures and images for: Hexose-6-phosphate dehydrogenase controls cancer cell proliferation and migration through pleiotropic effects on the unfolded-protein response, calcium homeostasis, and redox balance
Source: FASEB J. 2018 Jan 8;32(5):2690–705. doi: 10.1096/fj.201700870RR (PMC5901385; doi:10.1096/fj.201700870RR)

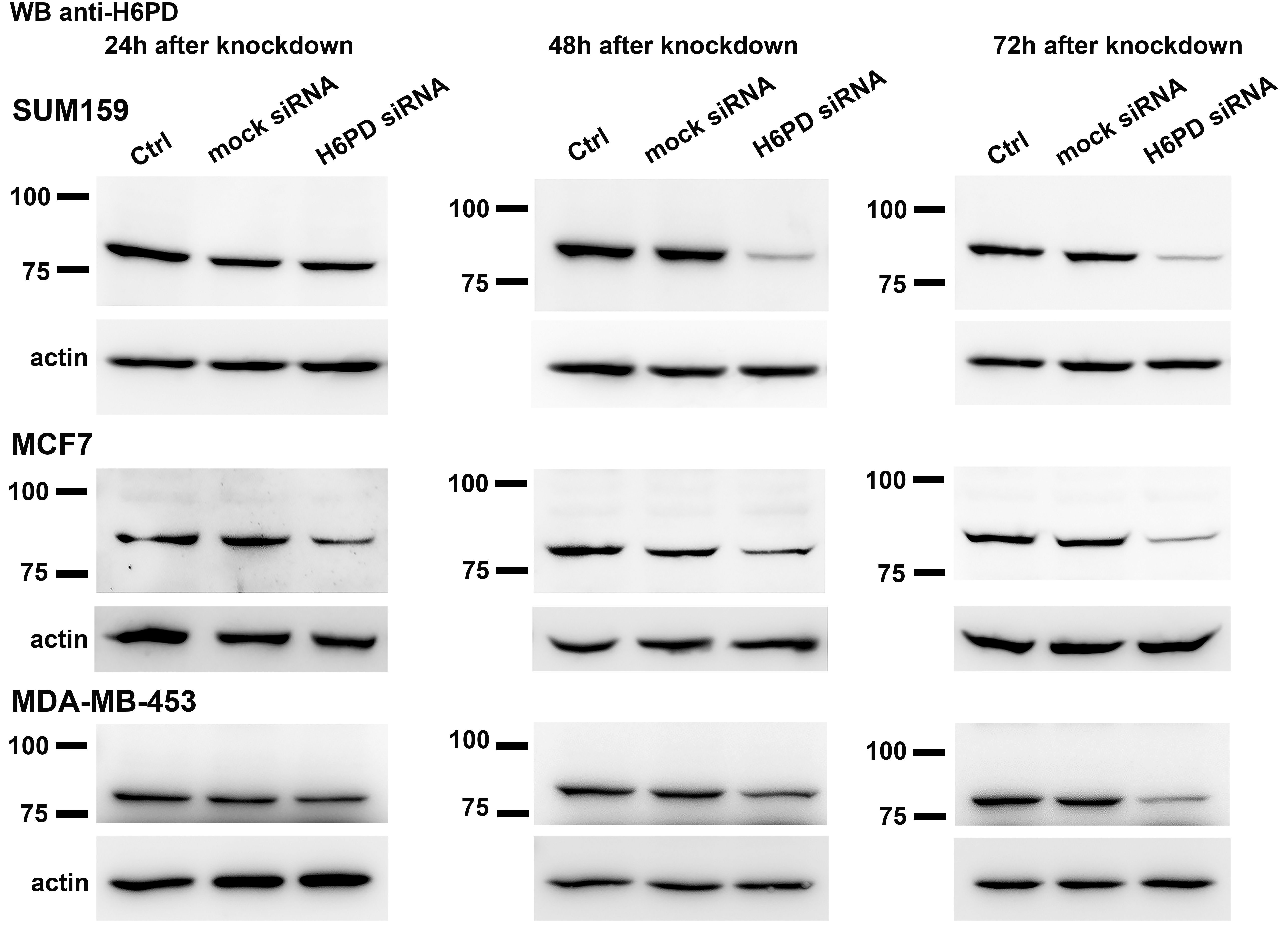

Supplement: Supplementary file 1 [file fj.201700870RR.sf1.tif]

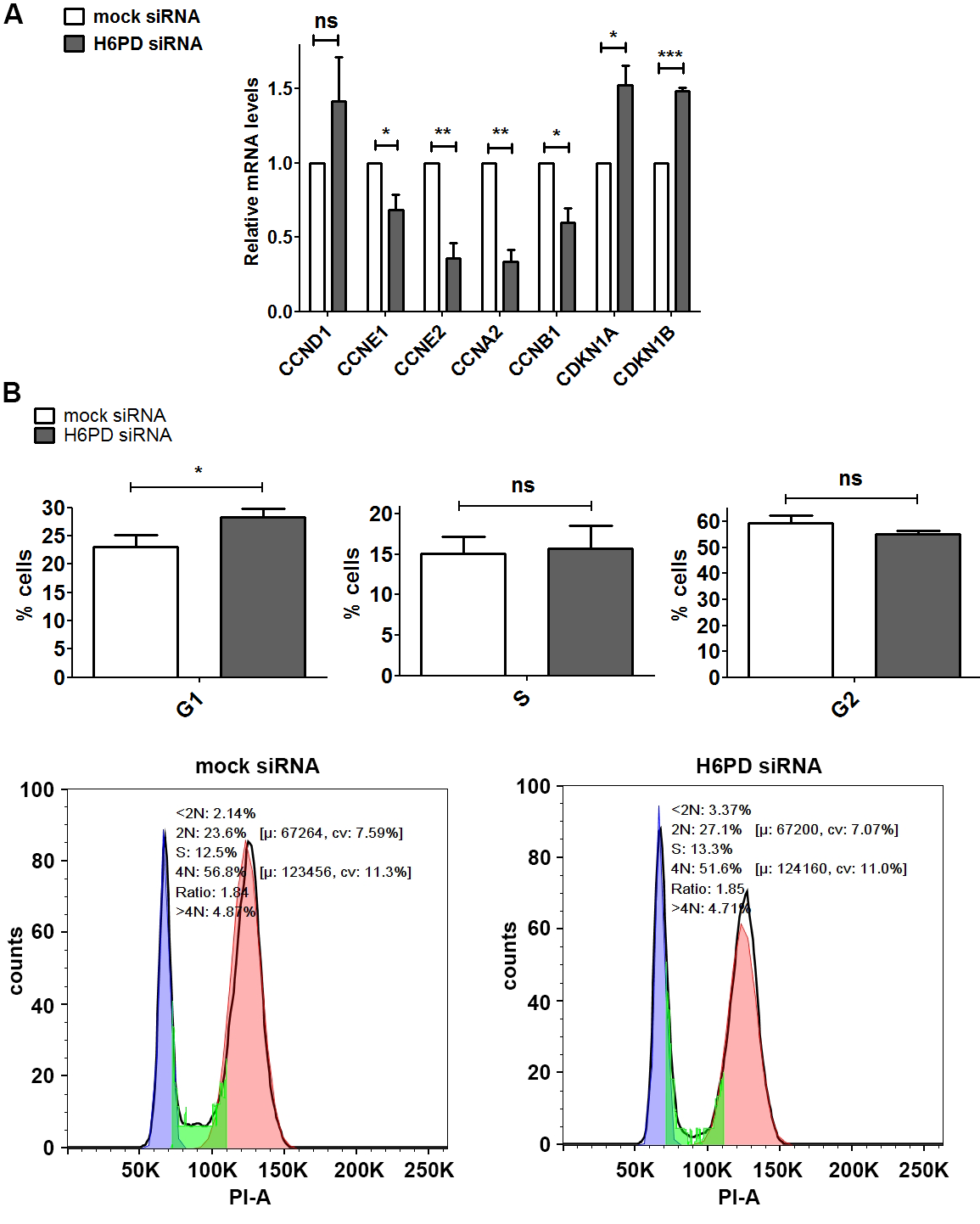

Supplement: Supplementary file 2 [file fj.201700870RR.sf2.tif]

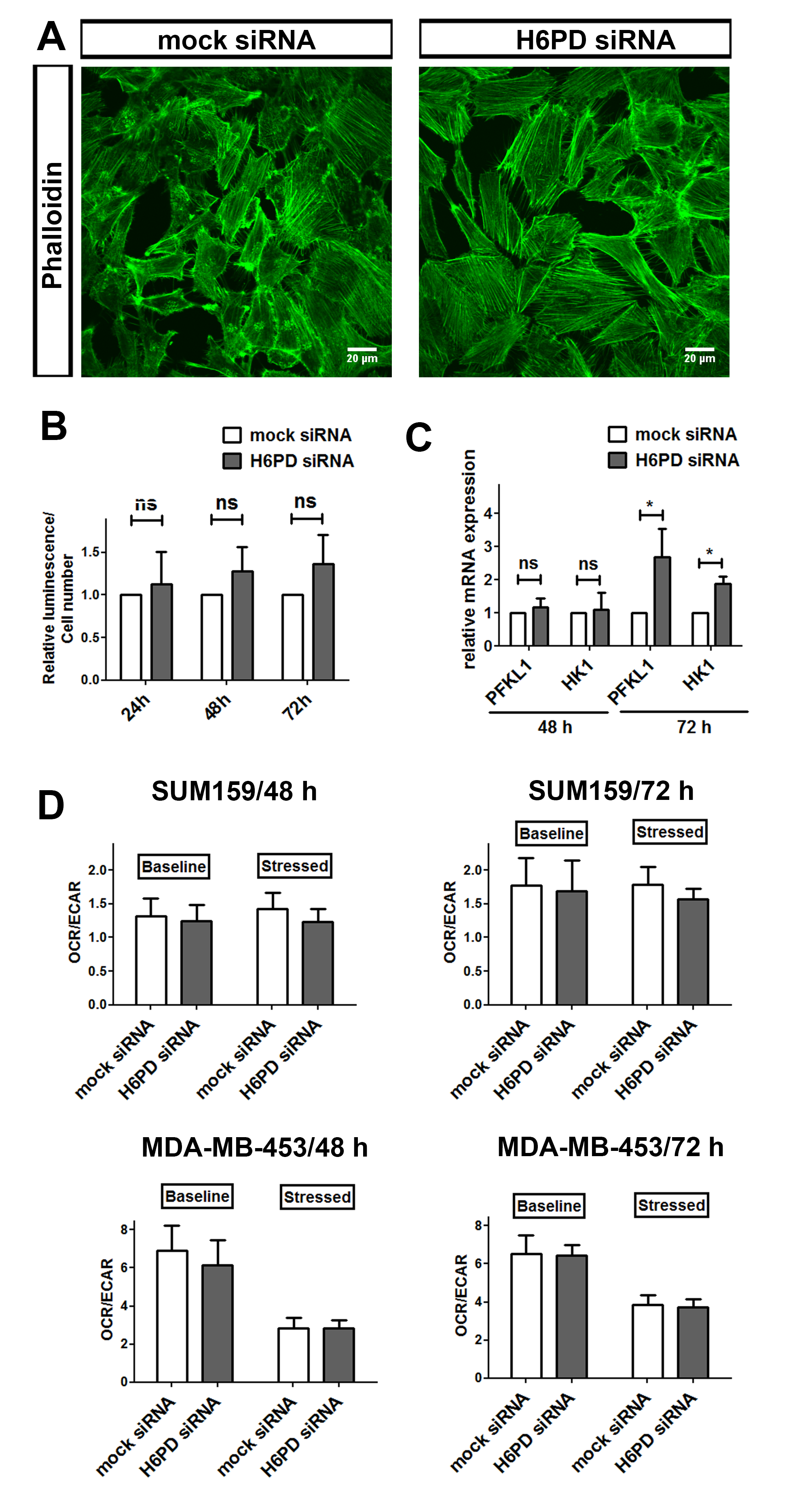

Supplement: Supplementary file 3 [file fj.201700870RR.sf3.tif]

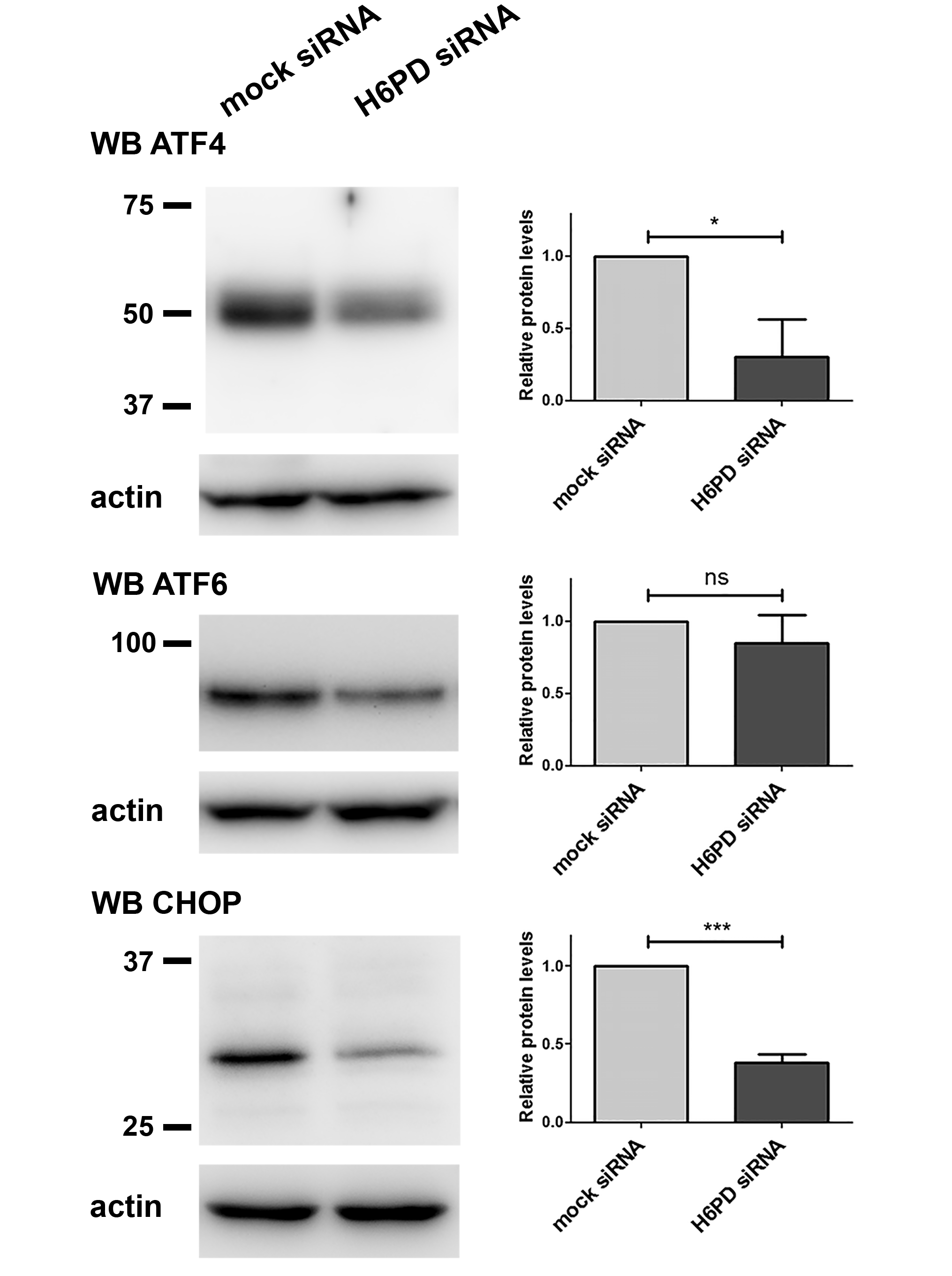

Supplement: Supplementary file 4 [file fj.201700870RR.sf4.tif]
