# Supplementary material for: Hexose-6-phosphate dehydrogenase controls cancer cell proliferation and migration through pleiotropic effects on the unfolded-protein response, calcium homeostasis, and redox balance
Source: FASEB J. 2018 Jan 8;32(5):2690–705. doi: 10.1096/fj.201700870RR (PMC5901385; doi:10.1096/fj.201700870RR)
Supplement: Supplementary file 5 [file fj.201700870RR.st1.docx]

**Supplemental Table 1. Sequence of primers used for qPCR.**

| **Gene name** | Primers (sense/antisense, 5’ to 3’ orientation) |
| --- | --- |
| *PPIA* | ATGGTCAACCCCACCGTGT/TCTGCTGTCTTTGGGACCTTGTC |
| *ATF4* | CCAAGCACTTCAAACCTCATG/ATCCATTTTCTCCAACATCCAATC |
| *ATF6* | CCTGTCCTACAAAGTACCATGAG/CCTTTAATCTCGCCTCTAACCC |
| *CHOP* | GTACCTATGTTTCACCTCCTGG/TGGAATCTGGAGAGTGAGGG |
| *MMP1* | GCACAAATCCCTTCTACCCG/TGAACAGCCCAGTACTTATTCC |
| *MMP2* | ACCCATTTACACCTACACCAAG/TGTTTGCAGATCTCAGGAGTG |
| *MMP14* | TGCCTACCGACAAGATTGATG/ATCCCTTCCCAGACTTTGATG |
| *Vim* | ACCCTGCAATCTTTCAGACAG/GATTCCACTTTGCGTTCAAGG |
| *CTNNB1* | GTTCAGTTGCTTGTTCGTGC/ GTTGTGAACATCCCGAGCTAG |
| *HK1* | ACATTGTCTCCTGCATCTCTG/ GCCTTAAAACCCTTTGTCCAC |
| *PFKL* | AACGAGAAGTGCCATGACTAC/GTCCCATAGTTCCGGTCAAAG |
| *CCND1* | CCTCGGTGTCCTACTTCAAATG/ GCGGTCCAGGTAGTTCATG |
| *CCNE1* | TCTTGAGCAACACCCTCTTC/ TTCTTGTGTCGCCATATACCG |
| *CCNE2* | AAGAGGAAAACTACCCAGGATG/ATAATGCAAGGACTGATCCCC |
| *CCNA2* | CCTTTCATTTAGCACTCTACACAG/ CCAGGGTATATCCAGTCTTTCG |
| *CCNB1* | GGCTTTCTCTGATGTAATTCTTGC/ GTATTTTGGTCTGACTGCTTGC |
| *CDKN1A* | TGTCACTGTCTTGTACCCTTG/GGCGTTTGGAGTGGTAGAA |
| *CDKN1B* | TCTGAGGACACGCATTTGG/TGTTCTGTTGGCTCTTTTGTTT |
