# Supplementary material for: Hexose-6-phosphate dehydrogenase controls cancer cell proliferation and migration through pleiotropic effects on the unfolded-protein response, calcium homeostasis, and redox balance
Source: FASEB J. 2018 Jan 8;32(5):2690–705. doi: 10.1096/fj.201700870RR (PMC5901385; doi:10.1096/fj.201700870RR)
Supplement: Supplementary file 6 [file fj.201700870RR.sd1.docx]

**Supplemental Figure 1**

**Supplemental Figure 1.** Efficiency of downregulation of H6PD expression with siRNAs. SUM159, MCF7 or MDA-MB-453 cells were left untreated (Ctrl), transfected with mock siRNA or siRNA against H6PD. Endogenous expression was assessed by western blotting using a specific anti-H6PD antibody at 24 h, 48 h and 72 h after siRNA delivery.

**Supplemental Figure 2.** *A)* Gene expression analysis with qPCR in the SUM159 cell line, 48 h after H6PD knockdown for the indicated genes. *B)* Cell cycle analysis 48 h after H6PD knockdown in the SUM159 cells, using propidium iodide staining and FACS. The average of three independent experiments for the G1/S/G2 phases is shown (top), as well as the measurement from one representative experiment (bottom).

**Supplemental Figure 3.** *A)* Phalloidin staining of SUM159 cells 72 h after transfection with mock or H6PD siRNAs. *B)* The CellTiter-Glo® Luminescent assay as a measure for ATP levels was employed in SUM159 cells at 24 h, 48 h or 72h after mock or H6PD siRNA delivery. In parallel, the cell number in each sample was assessed after nuclear staining using Hoechst-33342 and high-content imaging. The ratio of luminescence units to the total cell number counted from three independent experiments is depicted. *C)* qPCR analysis for mRNA levels of HK1 and PFKL in the SUM159 cell line, 48 h and 72 h after H6PD knockdown. *D)* Graphical representation of the OCR/ECAR ratios for all conditions described in Figure 7.

**Supplemental Figure 4.** Effect of H6PD knockdown on ATF4, ATF6 and CHOP expression 42 h after siRNA transfection. With the exception of the different time point in which samples were analyzed, the experiment was performed essentially as described in Figure 3.
